# Use of Thiopurines and Risk of Colorectal Neoplasia in Patients with Inflammatory Bowel Diseases: A Meta-Analysis

**DOI:** 10.1371/journal.pone.0081487

**Published:** 2013-11-28

**Authors:** Jianfeng Gong, Lijing Zhu, Zhen Guo, Yi Li, Weiming Zhu, Ning Li, Jieshou Li

**Affiliations:** 1 Department of General Surgery, Jinling hospital, Medical School of Nanjing University, Nanjing, PR China; 2 Department of Oncology, Drum Tower hospital, Medical school of Nanjing University, Nanjing, PR China; University of Chicago, United States of America

## Abstract

**Objective:**

Inflammatory bowel disease (IBD) is commonly treated with thiopurines such as azathioprine and mercaptopurine for the maintenance of remission. Studies examining chemopreventive of these medications on colorectal neoplasm in IBD patients have yielded conflicting results. We performed a meta-analysis to assess the role of thiopurines for this indication.

**Methods:**

We performed a systematic search of PubMed, Web of Science, EMBASE and Cochrane to identify studies reporting colorectal neoplasm from IBD patients treated with thiopurines and conducted a meta-analysis of pooled relative risk (RR) using the random effects model.

**Results:**

Nine case-control and ten cohort studies fulfilled the inclusion criteria. The use of thiopurines was associated with a statistically significant decreased incidence of colorectal neoplasm (summary RR=0.71, 95% CI=0.54–0.94, p=0.017), even after adjustment for duration and extent of the disease, but there was high heterogeneity among studies (*I* ^2^=68.0%, p<0.001). The RR of advanced neoplasm (high-grade dysplasia and cancer) was 0.72 (95%CI=0.50–1.03, p=0.070) and that of cancer was 0.70 (95% CI=0.46–1.09, p=0.111) for thiopurine-treated patients. Heterogeneity of the studies was affected by the sample size (</≥100 cases) and whether the patients had longstanding colitis (≥7 years).

**Conclusion:**

The current meta-analysis revealed that thiopurines had a chemopreventive effect of colorectal neoplasms and a tendency of reducing advanced colorectal neoplasms in IBD. Due to the heterogeneity of included studies, these results should be interpreted with caution.

## Introduction

The association of inflammatory bowel disease (IBD) and colorectal cancer (CRC) has been well documented. The presence of long-standing ulcerative colitis (UC) increased the risk of CRC with a cumulative risk approaching 2% after 2 years, 8% after 20 years, and 18% after 30 years of disease duration [1]. A similar CRC risk was observed in Crohn’s colitis based on population-based studies [2]. Usage of chemoprophylaxis agents, especially 5-aminosalicylic acids (5-ASA), has been suggested in prior studies [3]. However, a very recent meta-analysis yielded inconsistent results concerning 5-ASA that were dependent on the inclusion of either non-referral or clinic-based populations [4].

Thiopurines, including azathioprine (AZA) and its analogue, mercaptopurine (MP), are commonly used to treat IBD. AZA/MP is effective as steroid-sparing agents [5] and has been demonstrated to induce and maintain remission in patients with IBD [6]. However, thiopurines have been implicated in the development of malignancy due to its immunosuppressive and potential mutagenic effects, especially lymphoma [7] and skin cancers [8]. This often leads to a dilemma as the role of thiopurines in high-risk IBD patients for malignancy [9]. As colorectal dysplasia or cancer is supposed to be the consequence of chronic inflammatory status, chemoprevention of inflammation using AZA/MP could potentially reduce the risk of CRC in patients with IBD. But the conclusion has not been definitively established [10]. There are no randomized controlled trials and results from observational studies have been mixed.

To evaluate this question, we sought to conduct a meta-analysis of existing observational studies examining the effect of AZA/MP use on CRC and dysplasia in patients with IBD.

## Materials and Methods

### Search Strategy

Two independent reviewers (GJF and ZLJ) completed an online systematic search using MEDLINE and ISI Web of Science (Web of Knowledge), EMBASE, and Cochrane library to identify all articles published between 1960 and August 2013 assessing the effect of thiopurine on risk of CRC and dysplasia in patients with IBD. The following terms were used: (Dysplasia OR cancer OR carcinoma OR neoplasm OR neoplasia OR malignancy OR adenocarcinoma OR tumor) AND ("inflammatory bowel disease" OR "crohn's disease" OR "ulcerative colitis") AND (mercaptopurine OR azathioprine OR thiopurine OR thioguanine OR "purine analogs") AND (colon OR colonic OR colorectal OR rectal OR rectum). References from the bibliographies of related articles were crosschecked to find additional articles. All searches were updated to August 17, 2013. Our study was designed, conducted, and reported according to the standards of quality for reporting meta-analyses [11].

### Inclusion and Exclusion Criteria

The inclusion criteria of articles were that the studies: (1) cohort or case-control study which included patients with a diagnosis of IBD, CD and/or UC or IBD-U; (2) exposed patients received one of the thiopurine agents (ie, AZA, MP); (3) outcomes for thiopurine-exposed patients were compared to a reference group unexposed to thiopurine, (4) were designed to detect development of colorectal cancer or dysplasia as a predetermined endpoint (5). relative risk (RR) with corresponding 95% confidence interval (CI) was provided or could be calculated using the raw data presented in the studies. Two investigators (ZLJ and GZ) applied the inclusion criteria independently. When disagreements occurred, a senior author (ZWM) was referred to arrive at a consensus.

Studies that did not report outcomes for an IBD-only population or in which it was not possible to extract outcome data from published results were excluded. Case reports, series, review articles, comments, letters, animal experiment and *in vitro* studies were also excluded.

### Data Extraction

Data were extracted independently from each study by two authors (GZ, LY) and entered into an Excel database. The following items were extracted: first author, publication date, country of origin, study population, time period of study, type and dosage of drugs, treatment duration, follow-up, number of cases and controls, distribution of IBD diagnosis (UC *vs.* CD), duration of IBD, type of colorectal neoplasm, potential confounders used for adjustment, and RRs adjusted for potential confounders.

When possible, maximally adjusted RRs for colorectal neoplasm of individual studies were pooled. For studies that did not report RRs, 2×2 contingency tables were constructed based on reported outcomes and ORs were calculated using Mantel-Haenszel method.

### Quality Assessment

To assess the study quality, an evaluation system based on the Newcastle-Ottawa Scale was adopted (range 0-9 stars)[12]. The included studies were judged on 3 aspects: the selection of study populations, the comparability of the populations, and determination of exposure (case-control studies) or outcomes of interest (cohort studies), respectively. The full score was 9 stars, and a high-quality study was defined as a study with seven stars or more. A table containing the rankings for each study is shown in the Table S1 **.**

### Statistical Analysis

The association between thiopurine and colorectal neoplasm was expressed as relative risks (RRs) with 95% CIs, which represent the relative risk of CRC or dysplasia exposed to thiopurines compared to controls. Pooled RRs were calculated using the random effects model, which was used to account for variations between studies and give a more conservative pooled estimate.

Heterogeneity across studies was assessed using the *I* ^2^ statistic and the Cochran’s Q test, while *I* ^2^ >50% or Cochran’s Q <0.10 was considered to indicate significant heterogeneity across studies. In order to explore potential source of heterogeneity and evaluate different forms of possible bias, subgroup analysis were performed. These included subgroup meta-analysis based on study population (non-referral *vs.* clinic-based studies), type of relative risk estimation (odds ratio/risk ratio *vs.* hazard ratio), sample size (<100 *vs.* ≥100), longstanding IBD (≥7 years)( yes *vs*.no ), thiopurine usage >6m (yes *vs.* no), IBD type (UC only *vs.* mixed), geographic distribution of studies, mean/median follow-up period (>5 years) (yes *vs.* unknown), and whether or not the studies are adjusted for certain important potential confounders (5-ASA, age and sex, duration and extent of disease). No-referral study means that the outcome data was from population-based or geographically representative registries or databases, while clinic-based study only includes populations from gastroenterology clinics or IBD surveillance centers.

Two sensitivity analyses were used to test the robustness of our results. The first sensitivity analysis removed one study at a time to see if any single study was driving the results. The second sensitivity analysis included only studies with NOS quality score≥7. Funnel plots and the Egger’s test were used to evaluate for publication bias.

All statistical analyses were carried out with STATA, version 11.0 (Stata Corp, College Station, TX). *P*< 0.05 was considered statistically significant. All statistical tests were two-sided. For power analysis and sample size calculation, PASS software version 11.0 (NCSS, Kaysville, UT, USA) was used.

## Results

### Studies Retrieved

**Figure 1:**
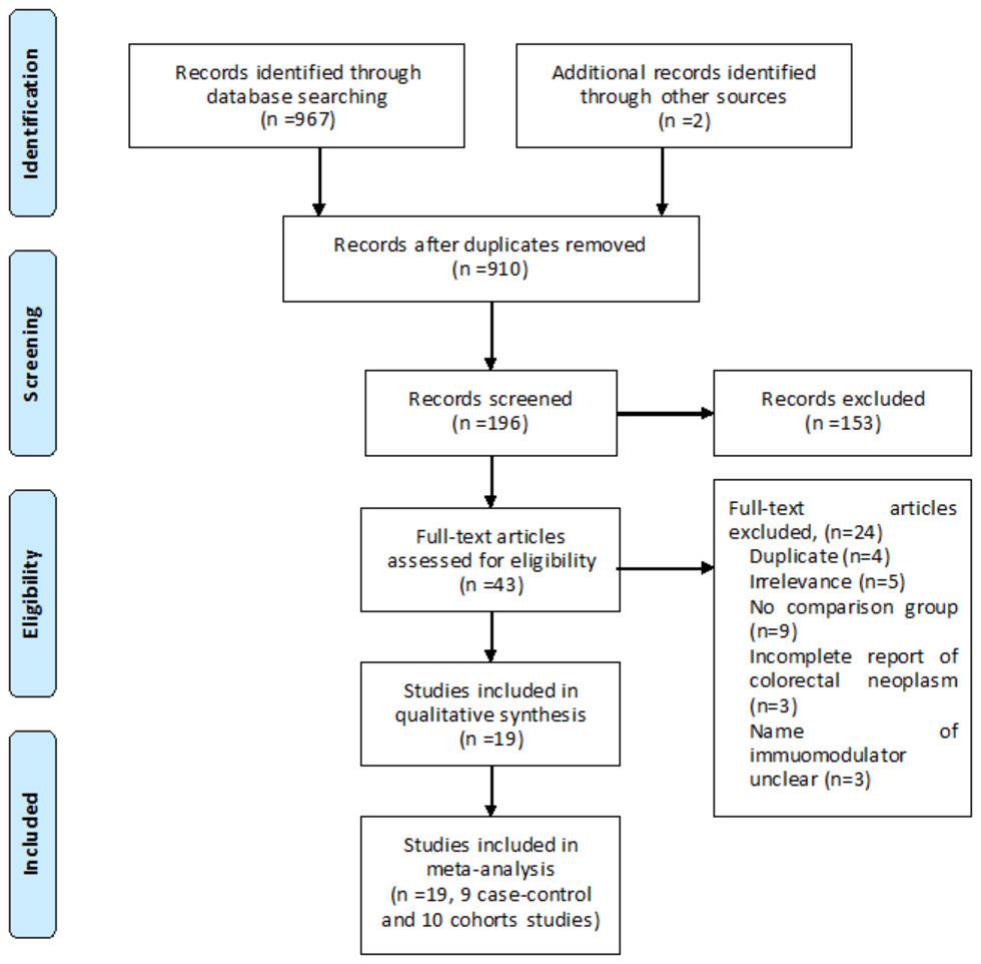
Search and study selection process.

We identified 969 titles and abstracts from electronic databases using the search algorithm. 926 were eliminated on review of titles and abstracts. Of the remaining 43 studies, 24 were excluded because of irrelevance (n=5), lack of control group (n=9), incomplete information on colorectal neoplasm (n=3)[13-15], name of immunomodulator unclear (n=3), or abstract fully published later (n=4). Thus, 19 full text articles were reviewed for meta-analysis. The study selection process is detailed in Figure **1**

### Study Characteristics

Of the 19 studies, nine were case-control studies [16-24] and 10 were cohort studies[25-34]. Four of the cohort studies calculated incidence rate as the measure of hazard ratio (HRs) [25,29,30,32] and one presented rate ratio (RR)[31]. In terms of geographical settings of the studies, 8 studies were conducted in the Unites States[18,20,22-24,29-31], 9 in Europe[16,17,19,21,25,26,28,31,33], one in New Zealand[27] and one in South Africa[34]. Two studies (Connell [26] & Rutter [21]) were from the same center (St’ Mark Hospital) but the overlap period was short (3 years), both studies were enrolled for analysis.

Nine studies reported CRC outcomes alone[16,17,22,24,26,28,31,32,34], one study reported dysplasia outcome alone[23], 3 reported CRC outcome and a combined CRC/dysplasia outcome[19,21,27], and the other 6 studies reported a combined outcome of CRC or dysplasia[18,20,25,29,30,33]. The characteristics of the included studies are outlined in Table **1**.

**Table 1:** Characteristics of included studies.

***Case-control studies***												
**Author, year**	**Drugs**	**Dosage**	**Duration**	**Follow up (y)**	**IBD type**	**Case/Ctrl**	**Neoplasm**	**OR/RR/HR(95%CI)**	**Covariates adjusted or matched**	**Duration of IBD (y)**	**Study type**	**NOS**
Baars (2011)[16]	Thio	n.a.	n.a	15.5	UC/CD/IBD-U	159/392	CRC	0.3(0.16-0.56)	2,3,8,9,10,14	9(1-23)	NR	5
Lakatos (2006) [17]	AZA	n.a.	n.a.	>1	UC	13/710	CRC	1.57(0.19-12.4)	Unadjusted	10(5-16)	NR	4
Lashner (1997) [18]	AZA /MP	n.a.	n.a.	6.3	UC	27/68	CRC/D	1.12(0.26-4.77)*	1,4,5,6,7	>8	CB	5
Nieminen (2013)[19]	Thio	n.a.	n.a.	n.a.	UC/CD	178/370 (all) & 31/159(CRC)	CRC/D	0.31(0.19-0.51)(any) 0.085(0.02-0.33)(CRC)	1,2, 3,4,5,8,9	12.2±10.2(case) vs.10.2±9.0(ctrl)	CB	7
Rubin (2013)[20]	AZA/MP	81.1mg	</≥2y	n.a.	UC	59/141	CRC/D	0.28(0.12-0.65)(all) 0.19(0.05-0.70)(<2y) 0.27(0.09-0.78)(≥2y)	1,3,4	18.8	CB	8
Rutter (2004)[21]	AZA	n.a	>1y	n.a.	UC	36/72 (all) &14/28(CRC)	CRC/D	0.35(0.11-1.11)(any) 0.22(0.03-1.87)(CRC)	1,2,3,4,11	>5 (5-55)	CB	8
Tang (2010)[22]	AZA/MP	n.a.	n.a.	10.7	UC/CD	18/30	CRC	0.38(0.04-3.72)	1,2,3,4,9,13	Unknown	NR	8
Tung (2001)[23]	AZA	n.a.	n.a.	n.a.	UC	26/33	D	0.68(0.147-2.6)	Unadjusted	Unknown	CB	4
Velayos (2006)[24]	AZA /MP	1.0-2.5mg/kg	>1y	n.a.	UC	188/188	CRC	3.0(0.7-13.6)	1,2,3,4,5,7,8,10,11,12,15,26,27	3-52	CB	8
***Cohort studies***												
**Author, Year**	**Drugs**	**Dosage**	**Duration**	**Follow up (y)**	**IBD type**	**Thio (Y/N)**	**Neoplasm**	**OR/HR/RR(95%CI)**	**Covariate adjusted/matched**	**Duration of IBD (y)**	**Study type**	**NOS**
Beaugerie (2013)[25]	AZA /MP	n.a.	n.a.	2.9(2.4-3.3)	UC/CD	5867/10810 (all) & 2841(longstaning)	HGD/CRC	0.62 (0.31-1.26)‡ # 0.28 (0.09-0.89)‡ (Longstanding)	1,2,3,4,9	8.2±8.2& Longstanding(>10y)	NR	8
Connell (1994)[26]	AZA	2mg/kg	2d-15y	9(0.04-29)	UC	86/180	CRC	1.13(0.46-2.77)	1,2,3,4	>10	CB	9
Fraser (2002)[27]	AZA	n.a.	1-222m	6.9±5.5	UC/CD	626/1578	CRC/D	0.86(0.43-1.73)(any) 0.76(0.33-1.79)(CRC)	Unadjusted	6.6	CB	7
Garcia (2013)[28]	AZA/MP	AZA(50-250mg)MP(25-150mg)	AZA(1-300m)MP(1-118m)	12.2±7.6	UC/CD	429/383	CRC	0.961(0.942-0.981)	Unadjusted	9.75±7.65	CB	7
Gupta (2007)[29]	AZA /MP	n.a.	n.a.	6.7(4.2-8.8)	UC	117/301	CRC/D	1.0(0.6-1.6)(any)‡ 0.8 (0.3-2.7)‡ (HGD/CRC)	Unadjusted	>7(11.1-22.9)	CB	7
Matula (2005)[30]	MP/AZA	121mg	>3m	7.9±3.4	UC	96/219	CRC/D	1.06(0.59-1.93)‡(any) 1.3(0.45-3.75)‡ (HGD/CRC)	Unadjusted	>7(18.2±8.9)	CB	7
Pasternak (2013)[31]	AZA	n.a.	>6m	7.9(3.5-12.0)	UC/CD	5197/38772	CRC	1.36(0.75-2.49)¶†	1,2,4,5,7,9,16,17,18,19,20,21,22,23	Unknown	NR	9
Satchi (2013)[32]	MP	50-100mg	n.a	>10	UC/CD	27/27	CRC	1.23(0.35-4.28)	1,2,3,4,5,9,12,15	>20 (mean 24.1)	CB	8
Schaik (2012)[33]	AZA /MP	≥50mg	>6m	>0.5	UC/CD/IBD-U	770/1808	HGD/CRC	0.1(0.01-0.75)‡§ 0.12(0.02-0.87) ‡ (>1y)	1,2,3,4,5,6,9,14,24,25	3.1	NR	8
Setshedi (2011)[34]	AZA /MP	n.a.	>6m	9.9(3.3-18.4)	UC/CD/IBD-U	123/836	CRC	0.27(0.016-4.53)	unadjusted	Unknown	CB	7

Abbreviations: n.a., not available; UC, ulcerative colitis; CD, Crohn’s disease; IBD-U, Inflammatory bowel disease undetermined; AZA, azathioprine; MP, mercaptopurine; CRC: colorectal cancer; D, dysplasia; HGD, high-grade dysplasia; OR: odds ratio; HR: hazard ratio; RR: rate ratio; NR, non-referral population; CB, clinic-based population.

**Covariates adjusted or matched**:1: age; 2: sex; 3:extent or location of disease; 4: duration of disease; 5: 5-ASA; 6: folic acid use; 7: glucocorticosteroid use; 8: primary sclerosing cholangitis; 9: type of IBD; 10: pesudopolyps; 11: surveillance colonoscopy;12: smoking; 13: race; 14: colonic surgery before diagnosis of IBD 15: family history of CRC;16: calendar year (in 2-year intervals); 17: socioeconomic class; 18: degree of urbanization;19: comorbidities; 20, history of intestinal surgery; 21: history of intestinal, rectal, or anal fistula, abscess, or fissure 22: IBD hospitalizations in the last year; 23: other immunomodulators(Methotrexate, cyclosporine, cyclophosphamide); 24: history of dysplasia; 25; calcium use; 26:asprin; 27: NSAID

* RR (Relative risk) ‡HR hazard ratio ¶RR (Rate ratio) †The rate ratio of former thiopurine users *vs.* nonusers was not analyzed (RR=0.71, 95% CI 0.34-1.46). # Patients who have discontinued thiopurine were not analyzed (HR=1.23[0.65-2.35], p=0.53). §For patients receiving AZA/MP for over 1year, the HR is 0.12(0.02-0.87)

### Any colorectal neoplasia

**Figure 2:**
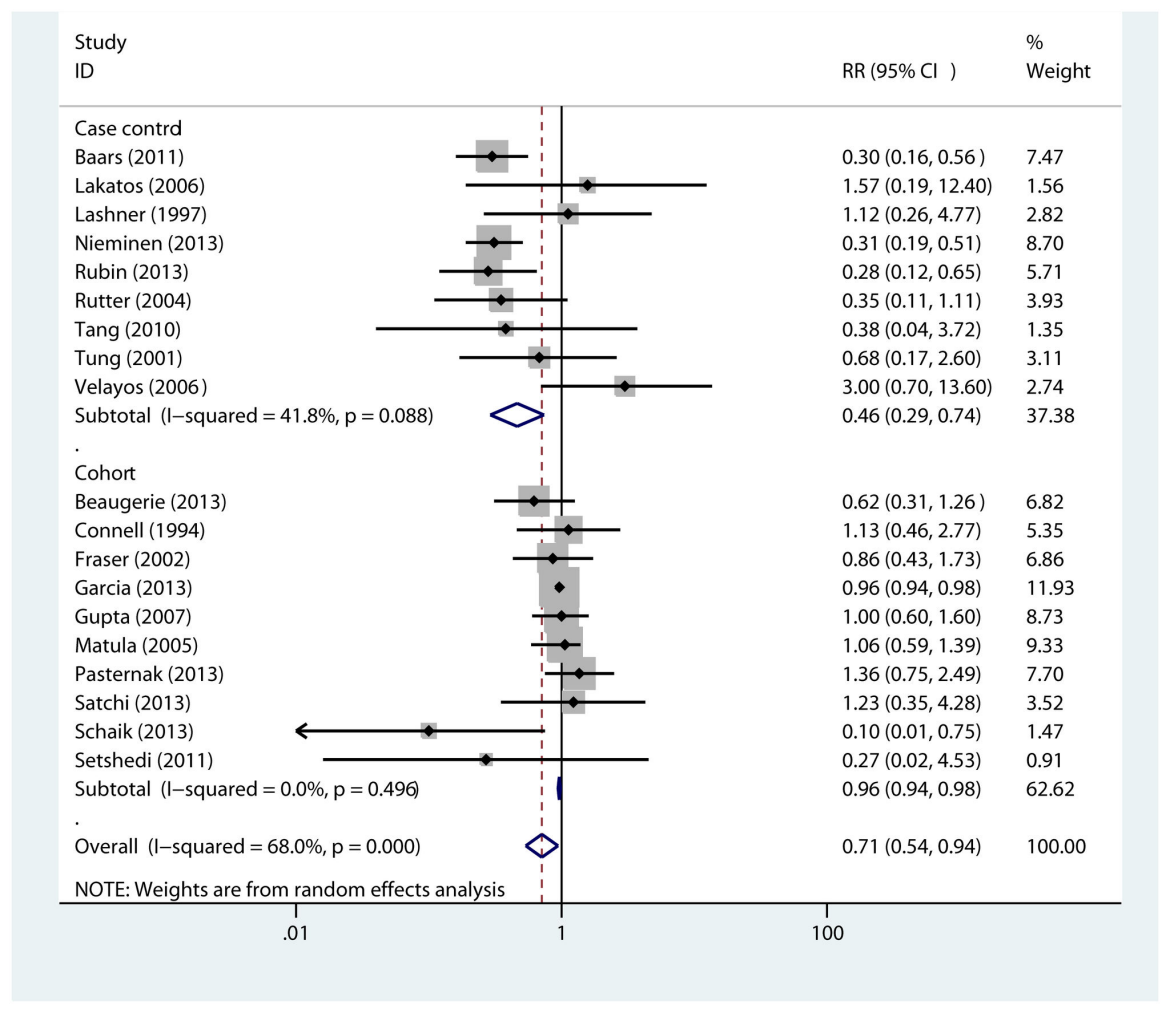
Association between thiopurine use and colorectal neoplasm risk in nine case-control and ten cohort studies. Random effects models were used as there were statistical heterogeneity across studies (*I* ^2^=68.0%, P <0.001). Usage of thiopurine is associated with decreased incidence of colorectal neoplasm in IBD patients (RR=0.71, 95% CI=0.54-0.94, P=0.017). RR: relative risk. CI: confidence interval.

All studies were pooled to assess the effect of thiopurine on a combined effect of CRC or dysplasia (high- and low-grade). As shown in Figure **2**, use of thiopurine was associated with a reduction in CRC or dysplasia in case-control studies [16-24](RR=0.46, 95% CI 0.29-0.74, p=0.001) and in cohort studies [25-34] (RR=0.96, 95% CI 0.94-0.98, p<0.001). There was heterogeneity for case-control (Q=13.76, *I* ^2^=41.8%, P=0.088) but not for cohort (Q=8.38, *I* ^2^=0.0%, P=0.496) studies. When case-control and cohort results were pooled together, the summary RR was 0.71 (95% CI 0.54-0.94, p=0.017, Q=56.22, *I* ^2^=68.0%, P value for heterogeneity <0.001).

### Advanced colorectal neoplasia

16 studies (6 case-controls [16,17,19,21,22,24] and 10 cohorts [25-34]) reported the effect of thiopurine on incidence of advanced colorectal neoplasm (CRC and high-grade dysplasia). Thiopurine usage prevented the occurrence of advanced neoplasia in cohort studies (RR 0.96, 95% CI 0.94-0.98, P<0.001, Q=8.79, P for heterogeneity 0.457, *I* ^2^=0.0%) but not case-control (RR 0.44, 95%CI 0.16-1.23, P=0.118, Q=14.38, P for heterogeneity=0.013, *I* ^2^=65.2%). Overall, the relative risk of advanced neoplasm is 0.72 (95%CI 0.50-1.03, P=0.070, Q=38.60, P for heterogeneity 0.001, *I* ^2^=61.1%) for thiopurine treated group versus non-treated group.

### Colorectal cancer

Use of thiopurine was associated with a reduction of CRC in cohort studies (n=6 [24,25,28-30,32], RR=0.96, 95%CI 0.94-0.98, P<0.01, Q=2.63, P for heterogeneity=0.756, *I* ^2^=0.0%) but not case-control studies (n=6[16,17,19,21,22,24], RR=0.43, 95% CI 0.17-1.08, P=0.072, Q=14.38, P for heterogeneity=0.013, *I* ^2^=65.2%). When pooled together, the relative risk is 0.70 (95%CI 0.46-1.09, P=0.111, Q=34.21, P for heterogeneity<0.001, *I* ^2^=67.8%).

### Sensitivity analysis

To explore the potential impact of any single study on outcome, one study was removed at a time on the first sensitivity analysis and the result did not change, suggesting that no single study drove the pooled RRs for CRC or dysplasia.

The second analysis, which analyzed studies with NOS≥7 (n=15 [19-22,24-34]. For random effect model: RR=0.75, 95%CI 0.56-1.00, P=0.051. For fixed effect model: RR=0.95, 95%CI 0.94-0.98, P<0.01, Q=42.52, P for heterogeneity <0.001, *I* ^2^=67.1%), showed results that were consistent with the overall pooled results.

### Subgroup analysis

We used various subgroup analyses to further evaluate several potential sources of heterogeneity. As shown in Table **2**, heterogeneity was not affected by study population, the type of RR estimation, duration of thiopurine therapy, IBD type, geographic distribution of the studies, long-term follow-up (>5years), and adjustment for confounding factors (sex, age, duration of disease, extent of disease, and usage of 5-ASA). However, sample size and whether the patients had longstanding IBD (≥7 years) had impact on study heterogeneity. The protective effect of thiopurine on colorectal neoplasm remained after adjustment for disease duration and extent (RR=0.54, 95% CI=0.32-0.92, P=0.023).

**Table 2:** Subgroup RR and 95% CI analysis of thiopurine usage and risk of colorectal neoplasm in patients with IBD (random-effect model).

	**No. of studies**	**Study heterogeneity**			**RR(95% CI)**	**P value**
		**Q**	**P-value**	***I*^2^**		
Study population						
Non-referral	6	15.43	0.009	67.60%	0.57(0.27-1.19)	0.133
Clinic-based	13	35.17	0	65.90%	0.76(0.56-1.04)	0.082
Type of relative risk estimation						
OR/RR	15	50.29	0	72.20%	0.68(0.47-1.00)	0.05
HR	4	5.8	0.122	48.30%	0.83(0.52-1.31)	0.417
Sample size						
Smaller (<100)	4	1.04	0.791	0.00%	0.89(0.43-1.86)	0.762
Larger (≥100)	15	55.14	0	74.60%	0.69(0.51-0.94)	0.019
Longstanding IBD(≥7yrs)						
Yes	6	4.98	0.418	0.00%	0.98(0.74-1.29)	0.863
No	13	54.16	0	77.80%	0.58(0.37-0.91)	0.017
Thiopurine therapy>6m						
Yes	6	17.85	0.003	72.00%	0.56(0.22-1.42)	0.221
no	13	36.59	0	67.20%	0.74(0.55-0.99)	0.044
IBD type						
UC only	9	13.94	0.083	42.60%	0.85(0.56-1.27)	0.413
Mixed	10	42.01	0	78.60%	0.61(0.39-0.95)	0.029
5-ASA usage adjusted/matched						
Yes	6	22.96	0	78.20%	0.81(0.34-1.94)	0.627
No	13	28.16	0.005	57.40%	0.71(0.54-0.94)	0.018
Sex/age adjusted/matched						
Yes	9	25.2	0.001	68.20%	0.69(0.39-1.29)	0.209
No	10	23.03	0.006	60.90%	0.74(0.54-1.02)	0.066
Disease duration/extent adjusted						
Yes	9	18.96	0.015	57.80%	0.54(0.32-0.92)	0.023
No	10	16.16	0.064	44.30%	0.89(0.69-1.14)	0.34
Sex/age/duration/extent of disease adjusted/matched						
Yes	8	17.26	0.016	59.50%	0.61(0.34-1.10)	0.098
No	11	24.33	0.007	58.90%	0.80(0.60-1.07)	0.127
Mean/median follow-up period						
>5yr	11	16.82	0.083	39.80%	0.90(0.72-1.14)	0.386
unknown	8	14.08	0.05	50.3	0.48(0.28-0.82)	0.007
Geographic distribution						
Europe	9	43.61	0	82.10%	0.60(0.37-0.97)	0.036
USA	8	11.64	0.113	39.80%	0.87(0.57-1.33)	0.529

### Publication bias

**Figure 3:**
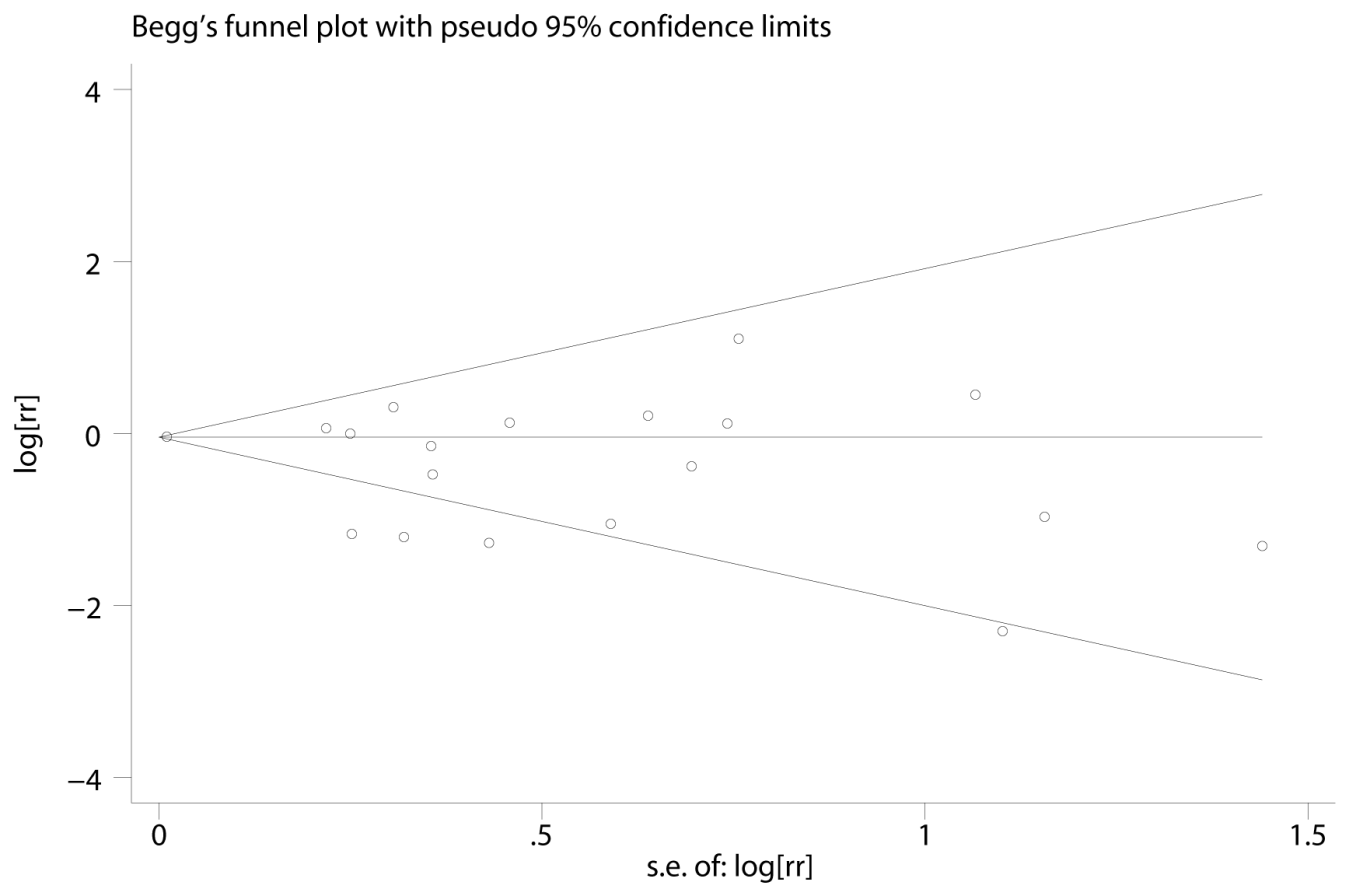
Funnel plots for overall studies. The shapes of the funnel plots did not reveal any evidence of obvious asymmetry visually. se: standard error; RR: Relative risk.

Visual inspection of the Begg’s funnel plot did not show significant asymmetry typically associated with publication bias (Figure **3**). Evidence of publication bias was also not significant with the Egger or Begg’s tests (P = 0.67 and 0.07, respectively).

### Sample size determination

Finally, we calculated the number of patients required to show a difference to develop a recommendation for an ideal study based on the findings of current meta-analysis. According to 9 case-control studies, the exposure to thiopurines in patients without colorectal neoplasia is 27.44% (550 of 2004 patients), while the incidence of colorectal neoplasia in thiopurine non-exposure patients is 1.00% (549 of 54613 patients) based on nine cohort studies (except for the study by Gupta et al [29]).

The optimal sample size calculation was based on the assumptions that the relative risk (RR) of colorectal neoplasia is 0.71 after thiopurine exposure. With the α error set at 0.05 and β error set at 0.10 (power of 90%), 1201 patients in each group would be required for a 1:1 matched case-control study. If a cohort study is to be carried out, 21511 patients in thiopurine exposure and non-exposure group would be needed, respectively.

## Discussion

This study provides a meta-analysis across studies to assess the risk of colorectal neoplasm from IBD on AZA or MP. Our results suggest thiopurine treatment has chemopreventive effect on colorectal neoplasm (defined as CRC and/or HGD and/or LGD) in IBD patients, and the result remained consistent after adjusting for the extent and duration of the disease. In addition, the use of thiopurines in patients with IBD might be associated with a tendency of reduction in the risk of advanced neoplasia (CRC and/or HGD) and colorectal cancer. We did not perform meta-analysis of the impact of thiopurine on dysplasia alone as there was only one study [23] reporting this issue.

Chemoprevention has been shown to reduce the risk of adenoma and CRC in general population[35], and there is increased interest in utilizing it in IBD patients recently[36]. Several agents have been studied in IBD including 5-ASA, corticosteroids, folic acid and ursodeoxycholic acid. However, at present, none have shown to have indisputable chemopreventive activity, including the most widely studied 5-ASA [4]. Thiopurines are already used as the cornerstone of maintenance therapy in as many as a third or more of IBD patients. But the effect of thiopurine on colorectal neoplasm is still controversial. Although in theory the immunosuppressive and potential mutagenic properties of thiopurine could contribute to the development of malignancies, especially non-melanoma skin cancer and lymphoma[37], in gastrointestinal tract, it also has chemopreventive effect by reducing inflammation and severity of disease activity, which might overweigh its carcinogenic effect. Our results showed for the first time in meta-analysis that thiopurine reduced the risk of colorectal neoplasm in IBD patients.

Based on two sensitivity analysis, our results are not changed by the influence of any single study or by study quality. Significant statistical heterogeneity was seen in our analyses, and to explore its origin, subgroup analyses were performed. Established risk factors for colorectal neoplasm in IBD include prolonged disease duration (>7-8 years) [38], male sex[39], extensive colonic involvement [40], family history of CRC [41], coexistence of primary sclerosing cholangitis (PSC) [42], active intestinal inflammation [43], and young age at disease onset [44]. Regular surveillance colonoscopy might reduce the risk [45]. Subgroup analysis of studies adjusted for these confounders revealed that heterogeneity was maximally eliminated when analyses were confined to longstanding colitis or studies with small samples (<100 patients). This suggests that the selection of the patients population in some studies might account for much of the heterogeneity observed. Adjustment for the extent and duration of the disease, however, did not change the statistical heterogeneity. Biological therapies, especially infliximab, are increasingly used in patients with IBD, and the concomitant usage of infliximab may have impact on azathiopurine metabolite [46,47]. However, we could not perform subgroup analysis of infliximab usage due to lack of sufficient data.

In the subgroup analysis, clinic based studies and non-referral studies were also analyzed separately, as meta-analysis may yield inconsistent results that were dependent on the population inclusion criteria. A very recent meta-analysis by Nguyen et al [4] revealed 5-ASA has no impact against CRC in IBD based on non-referral studies, in contrast, in clinic based studies, the effect is protective. As the majority of the clinic based studies were retrospective, the investigators may themselves be biased to the study hypothesis which may in turn results in selection bias. While non-referral data are more representative of the general population that ensures study findings more applicable to all IBD patients and circumvents concerns over generalizability. However, information on drug use and other confounding risk factors will be better recorded in clinical records than in large population based datasets where the variable data available is likely to be minimal and thus information on factors like extent of disease, severity, and medication use may be limited. In the current study, drug prescription data were not validated in 5 of 6 non-referral studies. Unfortunately, we did not find that different study population affects the result or heterogeneity of the meta-analysis.

There are several limitations to our analysis. First, because of the observational design, the interference of other potential factors could not be ruled out. They generally failed to account for one or more of the following factors associated with neoplasia risk: age, gender, duration and extent of disease, disease activity, family history of CRC, surveillance colonoscopy, smoking, use of 5-ASA and folic acid, and other potential factors. Secondly, protocols of the retrieved studies are not necessarily the same in terms of the doses and durations of the drugs and study period. The daily doses of AZA or MP and length of immunosuppressive therapy differed substantially among the studies (1 month to 19 years). Thiopurine acted slowly with the full therapeutic effect reached approximately 17 weeks after initiation, and greater efficacy has been demonstrated for increasing duration. Thirdly, patients who receive thiopurines are also likely to be those who are highest risk of CRC due to the severity of disease, and those who are not on thiopurines are likely to be on 5-ASAs, which might also be protective against CRC. This might blight the chemopreventive effect of thiopurine in some of the studies enrolled. To maximally circumvent this problem, we performed a subgroup risk assessment, and adjustment for duration and extent of disease strengthened the results of the study. Paradoxically, adjustment for 5-ASA use showed a statistically nonsignificant RR of 0.81.

Based on findings of the current meta-analysis, we could not design a potential therapeutic protocol of thiopurine in IBD patients. However, due to its chemopreventive effect on colorectal neoplasm, high risk patients such as those with a longer duration of colitis, early onset of disease, extensive colitis, more severe histological inflammation, family history of sporadic colorectal cancer, and co-existence with primary sclerosing cholangitis may benefit most from early and full dosage (for AZA, 2.0-2.5mg/kg.d, for MP, 1.0-1.5mg/kg.d) thiopurine therapy [48].

In summary, pooled results of existing observational studies suggested that thiopurine usage is preventive of colorectal neoplasia in IBD patients. However, the results of this meta-analysis should be interpreted with caution due to the heterogeneity of the studies included. To address this topic more explicitly, the optimal study would follow a large cohort of homogeneous IBD patients over a long period (e.g. at least 20 years) and would have the capacity to capture reliable data on medication usage and colorectal neoplasm outcome. This ideal study would be able to ascertain data on disease phenotype and duration in order to account for heterogeneity. The chemopreventive effect of thiopurines on colorectal neoplasm still warrants further investigation.

## Supporting Information

Table S1 **Methodological Quality of Case-control and Cohort Studies Included in the Meta-analysis.**
(DOCX)

Checklist S1 (DOC)

## References

[1] Eaden JA, Abrams KR, Mayberry JF (2001) The risk of colorectal cancer in ulcerative colitis: a meta-analysis. Gut 48: 526-535. doi:10.1136/gut.48.4.526. PubMed: 11247898. 11247898 PMC1728259

[2] Itzkowitz SH, Yio X (2004) Colorectal cancer in inflammatory bowel disease: the role of inflammation. Am J Physiol Gastrointest Liver Physiol 287: G7-G17. doi:10.1152/ajpgi.00079.2004. PubMed: 15194558. 15194558

[3] Levine JS, Burakoff R (2007) Chemoprophylaxis of colorectal cancer in inflammatory bowel disease: current concepts.Inflamm Bowel Dis 13: 1293-1298. doi:10.1002/ibd.20186. PubMed: 17567870. 17567870

[4] Nguyen GC, Gulamhusein A, Bernstein CN (2012) 5-aminosalicylic acid is not protective against colorectal cancer in inflammatory bowel disease: a meta-analysis of non-referral populations. Am J Gastroenterol 107: 1298-1304. doi:10.1038/ajg.2012.198. PubMed: 22751467. 22751467

[5] Chande N, Tsoulis DJ, Macdonald JK (2013) Azathioprine or 6-mercaptopurine for induction of remission in Crohn's disease. Cochrane Database Syst Rev 4:CD000545. 10.1002/14651858.CD000545.pub4 23633304

[6] Timmer A, McDonald JW, Tsoulis DJ, Macdonald JK (2012) Azathioprine and 6-mercaptopurine for maintenance of remission in ulcerative colitis. Cochrane Database Syst Rev 9:CD000478. PubMed: 2297204617253451. 10.1002/14651858.CD000478.pub3 22972046

[7] Kandiel A, Fraser AG, Korelitz BI, Brensinger C, Lewis JD (2005) Increased risk of lymphoma among inflammatory bowel disease patients treated with azathioprine and 6-mercaptopurine.Gut 54: 1121-1125. doi:10.1136/gut.2004.049460. PubMed: 16009685. 16009685 PMC1774897

[8] Peyrin-Biroulet L, Khosrotehrani K, Carrat F, Bouvier AM, Chevaux JB et al. (2011) Increased risk for nonmelanoma skin cancers in patients who receive thiopurines for inflammatory bowel disease. Gastroenterology 141: 1621-1628. doi:10.1053/j.gastro.2011.06.050. PubMed: 21708105. 21708105

[9] Dasari BV, McBrearty A, Gardiner K (2012) Immunosuppression in patients with Crohn's disease and neoplasia: an ongoing clinical dilemma. Dis Colon Rectum 55: 1008-1011. doi:10.1097/DCR.0b013e31825d9269. PubMed: 22874610. 22874610

[10] Devlin SM (2012) Do thiopurines prevent advanced colorectal neoplasia in patients with inflammatory bowel disease or is this an unanswerable question? Inflamm Bowel Dis 18: 1184-1185. doi:10.1002/ibd.21869. PubMed: 22271441. 22271441

[11] Stroup DF, Berlin JA, Morton SC, Olkin I, Williamson GD et al. (2000) Meta-analysis of observational studies in epidemiology: a proposal for reporting. Meta-Analysis of Observational Studies in Epidemiology (MOOSE). Group - JAMA 283: 2008-2012. doi:10.1001/jama.283.15.2008. 10789670

[12] Wells GA, Shea B, O'Connell D, Peterson J, Welch V et al. (2013) The Newcastle-Ottawa Scale (NOS) for assessing the quality of nonrandomized studies in meta-analyses. Avaiable at Available: http://www.ohri.ca/programs/clinical_epidemiology/oxford.asp (Accessed 7 April)

[13] Lichtenstein GR, Rutgeerts P, Sandborn WJ, Sands BE, Diamond RH et al. (2012) A pooled analysis of infections, malignancy, and mortality in infliximab- and immunomodulator- treated adult patients with inflammatory bowel disease. Am J Gastroenterol 107: 1051-1063. doi:10.1038/ajg.2012.89. PubMed: 22613901. 22613901 PMC3390465

[14] Armstrong RG, West J, Card TR (2010) Risk of cancer in inflammatory bowel disease treated with azathioprine: a UK population-based case-control study. Am J Gastroenterol 105: 1604-1609. doi:10.1038/ajg.2009.745. PubMed: 20104215. 20104215

[15] Biancone L, Zuzzi S, Ranieri M, Petruzziello C, Calabrese E et al. (2012) Fistulizing pattern in Crohn's disease and pancolitis in ulcerative colitis are independent risk factors for cancer: a single-center cohort study. J Crohns Colitis 6: 578-587. doi:10.1016/j.crohns.2011.11.005. PubMed: 22398047. 22398047

[16] Baars JE, Looman CW, Steyerberg EW, Beukers R, Tan AC et al. (2011) The risk of inflammatory bowel disease-related colorectal carcinoma is limited: results from a nationwide nested case-control study. Am J Gastroenterol 106: 319-328. doi:10.1038/ajg.2010.428. PubMed: 21045815. 21045815

[17] Lakatos L, Mester G, Erdelyi Z, David G, Pandur T et al. (2006) Risk factors for ulcerative colitis-associated colorectal cancer in a Hungarian cohort of patients with ulcerative colitis: results of a population-based study. Inflamm Bowel Dis 12: 205-211. doi:10.1097/01.MIB.0000217770.21261.ce. PubMed: 16534422. 16534422

[18] Lashner BA, Provencher KS, Seidner DL, Knesebeck A, Brzezinski A (1997) The effect of folic acid supplementation on the risk for cancer or dysplasia in ulcerative colitis. Gastroenterology 112: 29-32. doi:10.1016/S0016-5085(97)70215-4. PubMed: 8978339. 8978339

[19] Nieminen U, Jussila A, Nordling S, Mustonen H, Färkkilä MA (2014) Inflammation and disease duration have a cumulative effect on the risk of dysplasia and carcinoma in IBD: A case-control observational study based on registry data. Int J Cancer 2014;134: 189-196 PubMed: 23797639. 10.1002/ijc.28346 23797639

[20] Rubin DT, Huo D, Kinnucan JA, Sedrak MS, McCullom NE, et al (2013) Inflammation is an Independent Risk Factor for Colonic Neoplasia in Patients with Ulcerative Colitis: a Case-Control Study. Clin Gastroenterol Hepatol. 2013 Jul 17 pii: S1542-3565(13)01035-5 10.1016/j.cgh. [Epub ahead of print]. 10.1016/j.cgh.2013.06.023 PMC3840031 23872237

[21] Rutter M, Saunders B, Wilkinson K, Rumbles S, Schofield G et al. (2004) Severity of inflammation is a risk factor for colorectal neoplasia in ulcerative colitis. Gastroenterology 126: 451-459. doi:10.1053/j.gastro.2003.11.010. PubMed: 14762782. 14762782

[22] Tang J, Sharif O, Pai C, Silverman AL (2010) Mesalamine protects against colorectal cancer in inflammatory bowel disease. Dig Dis Sci 55: 1696-1703. doi:10.1007/s10620-009-0942-x. PubMed: 19705280. 19705280

[23] Tung BY, Emond MJ, Haggitt RC, Bronner MP, Kimmey MB et al. (2001) Ursodiol use is associated with lower prevalence of colonic neoplasia in patients with ulcerative colitis and primary sclerosing cholangitis. Ann Intern Med 134: 89-95. doi:10.7326/0003-4819-134-2-200101160-00008. PubMed: 11177311. 11177311

[24] Velayos FS, Loftus EV Jr, Jess T, Harmsen WS, Bida J et al. (2006) Predictive and protective factors associated with colorectal cancer in ulcerative colitis: A case-control study. Gastroenterology 130: 1941-1949. doi:10.1053/j.gastro.2006.03.028. PubMed: 16762617. 16762617

[25] Beaugerie L, Svrcek M, Seksik P, Bouvier AM, Simon T et al. (2013) Risk of Colorectal High-Grade Dysplasia and Cancer in a Prospective Observational Cohort of Patients with Inflammatory Bowel Disease. Gastroenterology;145: 166-175. doi:10.1053/j.gastro.2013.03.044. PubMed: 23541909. 23541909

[26] Connell WR, Kamm MA, Dickson M, Balkwill AM, Ritchie JK et al. (1994) Long-term neoplasia risk after azathioprine treatment in inflammatory bowel disease. Lancet 343: 1249-1252. doi:10.1016/S0140-6736(94)92150-4. PubMed: 7910274. 7910274

[27] Fraser AG, Orchard TR, Robinson EM, Jewell DP (2002) Long-term risk of malignancy after treatment of inflammatory bowel disease with azathioprine. Aliment Pharmacol Ther 16: 1225-1232. doi:10.1046/j.1365-2036.2002.01297.x. PubMed: 12144571. 12144571

[28] Gómez-García M, Cabello-Tapia MJ, Sánchez-Capilla AD, De Teresa-Galván J, Redondo-Cerezo E (2013) Thiopurines related malignancies in inflammatory bowel disease: Local experience in Granada, Spain. World J Gastroenterol 19: 4877-4886. doi:10.3748/wjg.v19.i30.4877. PubMed: 23946592. 23946592 PMC3740417

[29] Gupta RB, Harpaz N, Itzkowitz S, Hossain S, Matula S et al. (2007) Histologic inflammation is a risk factor for progression to colorectal neoplasia in ulcerative colitis: a cohort study. Gastroenterology 133: 1099-1105. doi:10.1053/j.gastro.2007.08.001. PubMed: 17919486. 17919486 PMC2175077

[30] Matula S, Croog V, Itzkowitz S, Harpaz N, Bodian C et al. (2005) Chemoprevention of colorectal neoplasia in ulcerative colitis: the effect of 6-mercaptopurine. Clin Gastroenterol Hepatol 3: 1015-1021. doi:10.1016/S1542-3565(05)00738-X. PubMed: 16234048. 16234048

[31] Pasternak B, Svanström H, Schmiegelow K, Jess T, Hviid A (2013) Use of Azathioprine and the Risk of Cancer in Inflammatory Bowel Disease. Am J Epidemiol 177: 1296-1305. doi:10.1093/aje/kws375. PubMed: 23514635. 23514635

[32] Satchi M, Korelitz BI, Panagopoulos G, Bratcher J, Yu C et al. (2013) Is treatment with 6-mercaptopurine for colitis associated with the development of colorectal cancer? Inflamm Bowel Dis 19: 785-788. doi:10.1097/MIB.0b013e318289664c. PubMed: 23392347. 23392347

[33] van Schaik FD, van Oijen MG, Smeets HM, van der Heijden GJ, Siersema PD et al. (2012) Thiopurines prevent advanced colorectal neoplasia in patients with inflammatory bowel disease. Gut 61: 235-240. doi:10.1136/gut.2011.237412. PubMed: 21602529. 21602529

[34] Setshedi M, Epstein D, Winter TA, Myer L, Watermeyer G et al. (2012) Use of thiopurines in the treatment of inflammatory bowel disease is associated with an increased risk of non-melanoma skin cancer in an at-risk population: a cohort study. J Gastroenterol Hepatol 27: 385-389. doi:10.1111/j.1440-1746.2011.06865.x. PubMed: 21793904. 21793904

[35] Arber N, Levin B (2008) Chemoprevention of colorectal neoplasia: the potential for personalized medicine. Gastroenterology 134: 1224-1237. doi:10.1053/j.gastro.2008.02.012. PubMed: 18395100. 18395100

[36] Subramanian V, Logan RF (2011) Chemoprevention of colorectal cancer in inflammatory bowel disease. Best Pract Res Clin Gastroenterol 25: 593-606. doi:10.1016/j.bpg.2011.09.003. PubMed: 22122774. 22122774

[37] Mason M, Siegel CA (2013) Do inflammatory bowel disease therapies cause cancer? Inflamm Bowel Dis 19: 1306-1321. PubMed: 23470503. 23470503 10.1097/MIB.0b013e3182807618

[38] Eaden JA, Abrams KR, Mayberry JF (2001) The risk of colorectal cancer in ulcerative colitis: a meta-analysis. Gut 48: 526-535. doi:10.1136/gut.48.4.526. PubMed: 11247898. 11247898 PMC1728259

[39] Jess T, Rungoe C, Peyrin-Biroulet L (2012) Risk of colorectal cancer in patients with ulcerative colitis: a meta-analysis of population-based cohort studies. Clin Gastroenterol Hepatol 10: 639-645. doi:10.1016/j.cgh.2012.01.010. PubMed: 22289873. 22289873

[40] Ekbom A, Helmick C, Zack M, Adami HO (1990) Ulcerative colitis and colorectal cancer. A population-based study. N Engl J Med 323: 1228-1233. doi:10.1056/NEJM199011013231802. PubMed: 2215606. 2215606

[41] Askling J, Dickman PW, Karlén P, Broström O, Lapidus A et al. (2001) Family history as a risk factor for colorectal cancer in inflammatory bowel disease. Gastroenterology 120: 1356-1362. doi:10.1053/gast.2001.24052. PubMed: 11313305. 11313305

[42] Torres J, Pineton de Chambrun G, Itzkowitz S, Sachar DB, Colombel JF (2011) Review article: colorectal neoplasia in patients with primary sclerosing cholangitis and inflammatory bowel disease. Aliment Pharmacol Ther 34: 497-508. doi:10.1111/j.1365-2036.2011.04753.x. PubMed: 21692821. 21692821

[43] Rutter M, Saunders B, Wilkinson K et al. (2004) Severity of inflammation is a risk factor for colorectal neoplasia in ulcerative colitis. Gastroenterology 126: 451-459. doi:10.1053/j.gastro.2003.11.010. PubMed: 14762782. 14762782

[44] Lutgens L, [!(surname)!], van Oijen MG, van der Heijden GJ, Vleggaar FP, Siersema PD et al. (2013) Declining risk of colorectal cancer in inflammatory bowel disease: an updated meta-analysis of population-based cohort studies. Inflamm Bowel Dis 19: 789-799. doi:10.1097/MIB.0b013e31828029c0. PubMed: 23448792. 23448792

[45] Karlén P, Kornfeld D, Broström O, Löfberg R, Persson PG et al. (1998) Is colonoscopic surveillance reducing colorectal cancer mortality in ulcerative colitis? A population based case control study. Gut 42: 711-714. doi:10.1136/gut.42.5.711. PubMed: 9659169. 9659169 PMC1727094

[46] Teichgräber U, Atreya I, Atreya R, Schwab M, Neurath MF (2013) Infliximab treatment induces levels of the active azathioprine metabolite TGTP in Crohn's disease. Inflamm Bowel Dis 19: E54-E55. doi:10.1097/01.MIB.0000438750.97521.d4. PubMed: 22532373. 22532373

[47] Wong DR, Pierik M, Seinen ML, van Bodegraven AA, Gilissen LP, et al (2013) The pharmacokinetic effect of adalimumab on thiopurine metabolism in Crohn's disease patients.J Crohns Colitis. Aug 6 PubMed: 23932783 pii: S1873-9946(13)00242-0 10.1016/j.crohns [Epub ahead of print]. PubMed: 23932783. 10.1016/j.crohns.2013.07.004 23932783

[48] Dyson JK, Rutter MD (2012) Colorectal cancer in inflammatory bowel disease: what is the real magnitude of the risk? World J Gastroenterol;18: 3839-3848. doi:10.3748/wjg.v18.i29.3839. PubMed: 22876036. 22876036 PMC3413056

