# Supplementary material for: Use of Thiopurines and Risk of Colorectal Neoplasia in Patients with Inflammatory Bowel Diseases: A Meta-Analysis
Source: PLoS One. 2013 Nov 28;8(11):e81487. doi: 10.1371/journal.pone.0081487 (PMC3842949; doi:10.1371/journal.pone.0081487)
Supplement: Table S1 — Methodological Quality of Case-control and Cohort Studies Included in the Meta-analysis. (DOCX) [file pone.0081487.s001.docx]

**Supporting Information**

**Table S1.** Methodological Quality of Case-control and Cohort Studies Included in the Meta-analysis

| Quality of Case-control Studies | | | | | | | | | |
| --- | --- | --- | --- | --- | --- | --- | --- | --- | --- |
| Author(year) | Adequate definition of cases | Representativeness of cases | Selection of controls | Definition of controls | Control for important factors ^a^ | Exposure ascertainment ^b^ | Same method of ascertainment for all subjects | Non-Response rate ^c^ | Total quality scores |
| Baars (2011) | ☆ | ☆ | ☆ | ☆ | .. | .. | ☆ | .. | 5 |
| Lakatos (2006) | ☆ | ☆ | .. | ☆ | .. | .. | ☆ | .. | 4 |
| Lashner (1997) | .. | ☆ | .. | ☆ |  | .. | ☆ | ☆ | 5 |
| Nieminen(2013) | ☆ | ☆ | ☆ | ☆ | ☆☆ | .. | ☆ | .. | 7 |
| Rubin(2013) | ☆ | ☆ | ☆ | ☆ | ☆ | ☆ | ☆ | ☆ | 8 |
| Rutter (2004) | ☆ | ☆ | ☆ | ☆ | ☆☆ | .. | ☆ | ☆ | 8 |
| Tang (2010) | ☆ | ☆ | ☆ | ☆ | ☆☆ | .. | ☆ | ☆ | 8 |
| Tung (2001) | .. | ☆ | .. | ☆ | .. | .. | ☆ | ☆ | 4 |
| Velayos (2006) | ☆ | ☆ | ☆ | ☆ | ☆☆ | .. | ☆ | ☆ | 8 |
| Quality of Cohort Studies | | | | | | | | | |
| Author(year) | Representativeness of exposed cohort | Selection of non- exposed cohort | Ascertainment of exposure | Outcome of interest not present at start of study | Control for important factors^d^ | Assessment of outcome | Follow-up long enough for outcomes to occur^e^ | Adequacy of follow up of cohorts ^f^ | Total quality scores |
| Beaugerie(2013) | ☆ | ☆ | ☆ | ☆ | ☆☆ | ☆ | .. | ☆ | 8 |
| Connell (1994) | ☆ | ☆ | ☆ | ☆ | ☆☆ | ☆ | ☆ | ☆ | 9 |
| Fraser (2002) | ☆ | ☆ | ☆ | ☆ | .. | ☆ | ☆ | ☆ | 7 |
| Garcia(2013) | ☆ | ☆ | ☆ | ☆ | .. | ☆ | ☆ | ☆ | 7 |
| Gupta (2007) | ☆ | ☆ | ☆ | ☆ | .. | ☆ | ☆ | ☆ | 7 |
| Matula (2005) | ☆ | ☆ | ☆ | ☆ | .. | ☆ | ☆ | ☆ | 7 |
| Pasternak (2013) | ☆ | ☆ | ☆ | ☆ | ☆☆ | ☆ | ☆ | ☆ | 9 |
| Satchi (2013) | ☆ | ☆ | ☆ | ☆ | ☆☆ | ☆ | .. | ☆ | 8 |
| Schaik (2013) | ☆ | ☆ | ☆ | ☆ | ☆☆ | ☆ | .. | ☆ | 8 |
| Setshedi (2011) | ☆ | ☆ | ☆ | ☆ | .. | ☆ | ☆ | ☆ | 7 |

^a^. maximum of 2 stars could be awarded for this item. Studies that controlled for age received one star, whereas studies that controlled for sex and duration of disease received an additional star.

^b^. studies that ascertained the expose with secure record or structured interview blind to case/control status received one star.

c. One star was assigned if there was no significant difference in the response rate between case and control subjects by using the chi-square test ( P > 0.05)

d. A maximum of 2 stars could be awarded for this item. Studies that controlled for age received one star, whereas studies that controlled for sex and duration of disease received an additional star.

e. A cohort study with a follow-up time > 5 years was assigned one star.

f. A cohort study with a follow-up rate > 80% was assigned one star
